# Development of Germinated-Brown-Rice-Based Novel Functional Beverage Enriched with γ-Aminobutyric Acid: Nutritional and Bio-Functional Characterization

**DOI:** 10.3390/foods13081282

**Published:** 2024-04-22

**Authors:** Rifat Jabeen, Nusrat Jan, Bazila Naseer, Prakash Kumar Sarangi, Kandi Sridhar, Praveen Kumar Dikkala, Maharshi Bhaswant, Syed Zameer Hussain, Baskaran Stephen Inbaraj

**Affiliations:** 1 Division of Food Science and Technology, Sher-e-Kashmir University of Agricultural Sciences and Technology of Kashmir, Srinagar 190025, India; 2 College of Agriculture, Central Agricultural University, Imphal 795004, India; 3 Department of Food Technology, Karpagam Academy of Higher Education Deemed to be University, Coimbatore 641021, India; 4 Department of Food Technology, Koneru Lakshmaiah Education Foundation Deemed to be University, Vaddeswaram 522502, India; dikkalapraveenkumar@gmail.com; 5 Center for Molecular and Nanomedical Sciences, Sathyabama Institute of Science and Technology, Chennai 600119, India; cmaharshi@gmail.com; 6 New Industry Creation Hatchery Center, Tohoku University, Sendai 9808579, Japan; 7 Department of Food Science, Fu Jen Catholic University, New Taipei City 242062, Taiwan

**Keywords:** germination, brown rice, γ-aminobutyric acid, functional beverage, storage

## Abstract

γ-aminobutyric acid (GABA), recognized as a primary inhibitory neurotransmitter within the brain, serves a crucial role in the aging process and in neurodegenerative conditions such as Alzheimer’s disease. Research has demonstrated the beneficial effects of GABA, particularly for elderly individuals. Given that elderly individuals often encounter challenges with swallowing food, beverages designed to address dysphagia represent a preferable option for this demographic. Among the different processing techniques, the germination process triggers biochemical changes, leading to an increase in certain nutrients and bioactive compounds (e.g., GABA). Therefore, we attempted to develop a novel functional beverage utilizing germinated brown rice enriched with GABA and studied its nutritional and bio-functional characterization. The optimal conditions (X_1_, X_2_, X_3_ and X_4._) were determined: powdered sugar (40 g), chocolate powder (20 g), sodium carboxymethyl cellulose (0.5 g), GBR (220 g), and water (440 mL). The results of storage studies indicated that the germinated-brown-rice-based beverage exhibited favorable nutritional attributes, including increased γ-oryzanol (52.73 ± 1.56%), total phenolic content (26.68 ± 1.56 mg GAE/100 g), niacin (5.17 ± 0.14%), and GABA (42.12 ± 0.63 mg/100 g) levels. Additionally, the beverage demonstrated notable antioxidant activity (74.23 ± 2.37 µmol TE/100 g), suggesting potential health-promoting effects. Sensory evaluation revealed satisfactory acceptability among consumers, highlighting its palatability. Overall, this study elucidates the development of a novel functional beverage utilizing germinated brown rice enriched with GABA, offering promising nutritional and bio-functional characteristics for health-conscious consumers.

## 1. Introduction

Nowadays, food serves not only to satisfy hunger, but also as a means of supplementing our diet for disease prevention and control. This has led to an increased demand for functional food that not only satisfies human hunger, but also provides immunological benefits to consumers [1]. Wholesome foods are essential to meet the public’s need for healthy nutrition. While milk- and fruit-based drinks are popular in the market and offer numerous health benefits such as providing essential vitamins and minerals, dairy products may pose issues like lactose intolerance and high cholesterol and saturated fatty acid content [2]. To address these concerns, cereal-based beverages and foods are emerging as promising alternatives. Cereal-based beverages, particularly those derived from rice, are preferred due to their rich dietary fiber and mineral content [3].

Rice (*Oryza sativa* L.) is a globally consumed cereal, but it is predominantly eaten as white rice. Brown rice (BR) is known for its nutritional richness, containing phytochemicals like γ-aminobutyric acid (GABA), ferulic acid, and γ-oryzanol, primarily found in its bran and germ layers [4]. However, BR consumption is restricted because of its cooking limitations, unappealing texture, and appearance [5]. The germination of BR has been explored as a cost-effective method to ameliorate its organoleptic qualities, texture, nutrient content, biological activity, and phytochemical bioavailability [6]. Germinated brown rice (GBR) has garnered attention for its biologically active compounds like GABA and γ-oryzanol, which have been associated with various health-promoting effects including stress reduction, the inhibition of cancer cell proliferation, and blood pressure control [7,8]. GBR is increasingly incorporated into a variety of food products such as bakery goods and salads, offering functional benefits [7,9]. The development of GABA-rich beverages from GBR presents an opportunity to create novel functional foods with potential market growth in the dietary supplement sector. Additionally, storage is an important stage that must be effectively monitored because numerous physicochemical and physiological changes occur during storage, significantly impacting the final product quality. Packaging techniques, storage duration, and storage temperature can all influence the stability of bioactive phytochemicals [10]. However, limited investigation has been conducted on the development of GABA-rich beverages derived from GBR. In light of these considerations, the goal of this research was to develop a GABA-rich beverage derived from GBR and assess its physicochemical properties, bioactive compounds, antioxidant activities, and sensory attributes during storage.

## 2. Materials and Methods

### 2.1. Raw Material

A commercial rice variety (Jhelum) commonly cultivated in temperate conditions in Northern Highland Himalayan areas was provided by the Mountain Research Centre for Field Crops, Khudwani, Sher-e-Kashmir University of Agricultural Sciences and Technology, Kashmir, (SKUAST-K), India. Simultaneously, a laboratory de-husker was used for the preparation of brown rice after the removal of the husk. 

### 2.2. Preparation of Germinated Brown Rice

Brown rice germination was conducted following our established protocol [5]: BR samples were sterilized with 0.1% sodium hypochlorite for 30 min, soaked in water for 5.76 h, and then placed in a germinator at 35 °C for 40 h on trays lined with moist filter paper. The relative humidity during germination was maintained at 70%. After germination, the grains were dried at 50 ± 5 °C in a tray dryer, milled, and sieved to produce germinated brown rice flour.

### 2.3. Experimental Design and Process Optimization

A Central Composite Rotatable Design (CCRD) was utilized to design the experiments employing Response Surface Methodology (RSM). The design incorporated both central and axial points and featured powdered sugar, sodium carboxymethyl cellulose, chocolate powder, and GBR/water as independent factors, while overall acceptability and total soluble solids were dependent responses. The ranges and coded levels of the independent variables are outlined in Table 1, and Design Expert^®^ 12 Stat Ease was employed to formulate second-order polynomial models for each response based on the experimental data. Multiple regression models were employed for data analysis, and analysis of variance (ANOVA) was utilized to ascertain the statistical significance of each outcome. A second-order polynomial model was applied to all parameters, as demonstrated in formula (1).

〖y〗 = b_0_ + ∑(i = 1)^4^ 〖b_i_ X_i_〗 + ∑(i = 1)^4^ 〖b_ii_ X^2^_i_〗 + ∑(i < j = 1)^4^ 〖b_ij_ X_i_ X_j_〗 (1)

where X_i_ (i = 1, 2, 3 and 4), X^2^_i_, and X_i_, X_j_ represent the linear, quadratic, and interaction effects of the independent variables on the response y, while b_0_, b_i_, b_ii_, and b_ij_ stand for the regression coefficients representing the intercept, linear, quadratic, and interactive effects, respectively. The optimum condition parameters utilized for numerical optimization aimed to maximize the total soluble solids and overall acceptability.

### 2.4. Preparation of GABA-Rich Beverage

**Figure 1:**
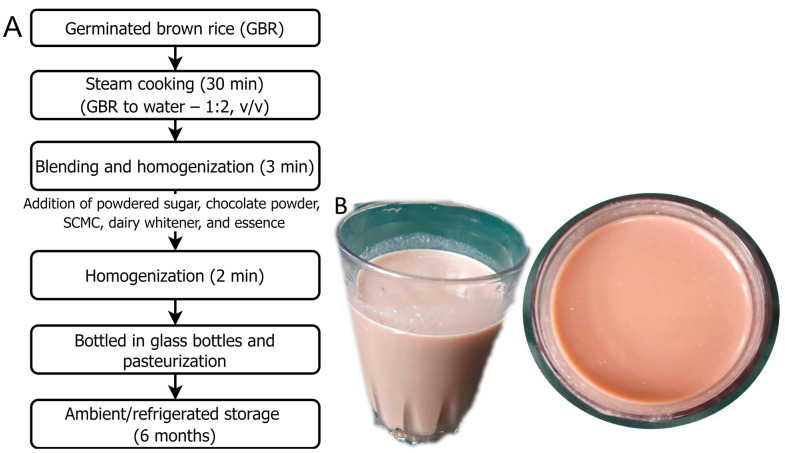
Preparation of germinated brown rice beverage (**A**) and optimized GABA-rich functional beverage from germinated brown rice (**B**). GBR, germinated brown rice; SCMC, sodium carboxymethyl cellulose.

To prepare a GABA-rich beverage, GBR was mixed with distilled water in the ratio of 1:2 (*v*/*v*) as per the experimental design (Table 1). This was followed by cooking in a steamer for 30 min. The cooked samples were then placed in a blender with water and the samples were homogenized for 3 min. Powdered sugar, chocolate powder, and sodium carboxymethyl cellulose (SCMC) were added to the slurry in different formulations as per the experimental design (Table 1). Additionally, dairy whitener (20 g) and essence (1–2 drops) were incorporated to enhance the mouthfeel, texture, flavor, appeal, and overall consumer acceptability. The mixture was homogenized again for 2 min. The beverage was then hot-filled in pre-sterilized glass bottles (cap. 220 mL) and capped immediately to prevent the access of air oxygen. Following this, pasteurization was performed at 85 °C for 15 min corresponding to 522.375 pasteurization units (Figure 1A). The beverage was then stored over a period of 6 months. A visual observation of the GABA-rich beverage is shown in Figure 1B. Furthermore, the GABA content in the GBR was found to be 48.18 mg/100 g, as per our previous studies [10].

#### 2.4.1. Overall Acceptability (OAA)

To evaluate the sensory properties, a semi-trained panel consisting of 30 individuals aged between 20 and 45 was selected to conduct acceptance tests for the GABA-rich beverage. The aim of the tests was to assess the level of preference using a 5-point hedonic rating scale, which measures the degree of liking or disliking.

#### 2.4.2. Total Soluble Solids (TSSs)

An analysis of the total soluble solids of the developed functional beverage was performed using a refractometer (Atago RX-1000) in accordance with the AOAC (2005). Briefly, the sample (1 g) was macerated with distilled water (1 mL) and the resulting mixture was placed on the refractometer. The results were given in degrees Brix.

### 2.5. Shelf-Life Evaluation

The GABA-rich functional beverage developed under optimized conditions was chosen for shelf life analysis and assessed for the proximate composition, total and reducing sugars, γ-aminobutyric acid, γ-oryzanol, niacin, total phenolics, antioxidant activities, and sensory attributes over a period of 6 months of storage (0, 2, 4, and 6) under ambient temperature (25 °C) and in refrigerated conditions (4 °C). The samples were withdrawn at regular intervals. 

#### 2.5.1. Proximate Composition

Gravimetric (925.10), Soxhlet (920.85), and Kjeldahl (920.87) techniques were employed for the estimation of moisture, fat, and protein contents, while fiber and ash contents were determined through sample digestion (962.09) and burning (923.03) using the standard AOAC techniques [11]. Carbohydrate (CHO) content was computed by the difference method, as given in formula (2) below.

CHO (%) = 100 − (%Moisture + %Fat + %Protein + %Fibre + %Ash)(2)

#### 2.5.2. Total and Reducing Sugars

The analysis of the total sugars was performed using the phenol sulfuric acid technique [12], while the standard protocol of Somogyi [13] was employed for the estimation of the reducing sugars in the GABA-rich beverage.

#### 2.5.3. γ-Oryzanol and GABA

The technique outlined by Moongngarm and Saetung [14] was adopted to assess γ-oryzanol contents, while the quantification of the GABA content in the functional beverage was carried out through high-performance liquid chromatography (HPLC) (YounglinR 930D) following the protocol outlined by Hussain et al. [5].

#### 2.5.4. Niacin Content

The niacin content of the GABA-rich beverage was checked by HPLC (YounglinR 930D) following the protocol of Moongngarm and Saetung [14].

#### 2.5.5. Total Phenolics and Antioxidant Capacities

The methanolic extracts were utilized to assess the total phenolic content using the Folin–Ciocalteu colorimetric method [10]. The ferric-reducing antioxidant power (FRAP) assay was employed using the standard protocol outlined by Queiros et al. [15], while the 1,1-diphenyl-2-picrylhydrazyl (DPPH) assay followed the technique described by Brand-Williams et al. [16]. 

#### 2.5.6. Total Plate Count

The total plate count of the functional beverage was illustrated using the protocol described by Jabeen et al. [17].

#### 2.5.7. Sensory Evaluation

The sensory characteristics of the GABA-rich beverage samples were evaluated by a group of thirty semi-trained panelists. A 50 mL sample in a glass container, duly coded with a three-digit code, was given to each of the panelists for evaluating the sensory attributes like flavor, color, acidity, and overall acceptability of the beverage using a five-point hedonic scale. Informed consent was obtained from all subjects involved in the sensory study.

### 2.6. Statistical Analysis 

The findings were expressed as mean ± standard deviation and the analyses were performed in triplicate. Statistical analysis was conducted using one-way ANOVA via SPSS statistical software (IBM Statistics 21.0, Chicago, IL, USA), with a significance level set at *p* < 0.05.

## 3. Results

### 3.1. Model Fitting

Table 2 displays the ANOVA results for the models developed for overall acceptability (OAA) and total soluble solids (TSSs), and indicates that the independent variables significantly affected these responses (*p* < 0.0001). The statistical analysis confirmed that the predicted models for OAA and TSSs were acceptable, with high R^2^ values. Furthermore, no significant lack of fit was observed for either response. The R^2^ values for OAA and TSSs were found to be 0.9596 and 0.9788, respectively. The predicted R^2^ and adjusted R^2^ values were also in good agreement for all significant models.

### 3.2. Overall Acceptability (OAA) 

Consumer perception towards the production of novel food products is a key element because it affects consumers’ desire to purchase them. A hedonic scale sensory analysis was carried out to measure the acceptability of the GABA-rich functional beverage prepared from GBR. Table 3 shows the average values of OAA ranging from 1.5 to 4.6. Equation (3) displays the predicted model for OAA in coded levels.

OAA = 3.15 + 0.9458X_1_ − 0.2292X_2_ (3)

**Figure 2:**
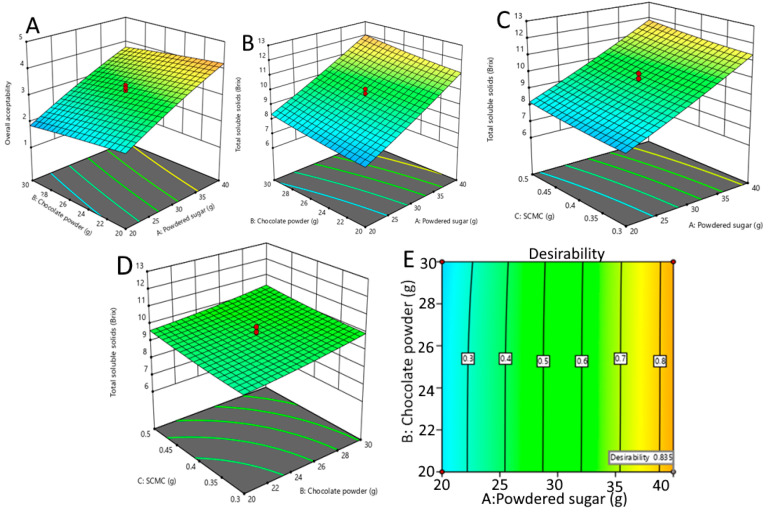
Effect of independent variables on dependent variables of GABA-rich functional beverage. Powdered sugar and chocolate powder on OAA (**A**), powdered sugar and chocolate powder on TSSs (**B**), powdered sugar and SCMC on TSSs (**C**), chocolate powder and SCMC on TSSs (**D**), and response surface plot showing desirability function (**E**). SCMC, sodium carboxymethyl cellulose; GABA, γ-aminobutyric acid; OAA, overall acceptability; TSSs, total soluble solids.

From the model of OAA, the positive linear effect of sugar (X_1_), and the negative linear effect of chocolate powder (X_2_) were significant (*p* < 0.0001). Therefore, an increased sugar concentration enhances the OAA and chocolate powder reduces the OAA of the GBR-based beverage (Figure 2A). The reason behind the increased acceptability of the beverage prepared from GBR with an increased sugar concentration and decreased acceptability with the addition of chocolate powder may be attributed to the taste and flavor preferences of consumers. GBR has a unique nutty and earthy flavor that may not blend well with the strong and bitter taste of chocolate. Additionally, sugar is known to enhance the overall taste and palatability of food and beverages, leading to increased consumer preference and acceptability [18]. Furthermore, the minimum OAA score was observed at experimental run no. 1 (1.5), where a lesser amount of sugar (10 g) was used. Experimental run no. 16 produced the highest OAA score of 4.6. The lesser content of sugar with a lesser and greater content of chocolate powder resulted in a lower OAA score. On the other hand, using the maximum concentration of chocolate powder had a negative impact on OAA and led to a lower score. Thus, the developed GABA-rich beverage (experiment 16) was highly accepted by the panelists and received all attribute scores. The hedonic ratings obtained for the functional beverage are similar to those previously investigated for non-fermented products obtained from oat, soy, coconut, rice, quinoa, and chickpeas [19,20,21].

### 3.3. Total Soluble Solids (TSSs)

Table 3 presents the average TSSs values that varied between 6.8 and 12.9 °Brix. The developed predicted model for TSSs in terms of coded levels is demonstrated by Equation (4).

TSS = 9.62 + 1.83X_1_ + 0.4167X_2_ + 0.2500X_3_ (4)

From the TSS model, the positive linear effects of sugar (X_1_), chocolate powder (X_2_), and SCMC (X_3_) were significant (*p* < 0.0001). Therefore, increased sugar concentration, chocolate powder, and SCMC enhance the TSSs of the functional beverage made from GBR (Figure 2B–D). The reason behind the enhancement of TSSs in the functional beverage prepared from GBR due to the increased levels of sugar, chocolate powder, and SCMC may be due to their ability to increase the viscosity and texture of the beverage. Sugar and SCMC are known to increase the viscosity and texture of food and beverages by increasing the concentration of dissolved solids. Additionally, chocolate powder contains cocoa solids, which are rich in polyphenols and have the ability to interact with the other components of the beverage, further contributing to its viscosity and texture [22].

### 3.4. Optimization and Validation

This study aimed to determine the optimal conditions for the production of a GABA-rich functional beverage that would result in the maximum levels of OAA and TSSs. The predicted conditions showed that the optimum values of OAA and TSSs were 4.27 and 11.54 °Brix, respectively. The optimal conditions were achieved using 40 g sugar, 20 g chocolate powder, 0.5 g SCMC, and 220 g of GBR/440 mL water. The graphical representation in Figure 2E illustrates that the optimal conditions were achieved, yielding the highest desirability value of 0.835 using the desirability function approach. The experimental values for OAA and TSSs were close to the predicted values with a difference of less than 4%, indicating the accuracy and reliability of the models.

### 3.5. Physicochemical Analysis

Table 4 and Table 5 display the changes noticed in the physicochemical characteristics of the GABA-rich functional beverage over a storage period of 6 months, both under ambient and refrigerated conditions. The findings indicated that a significant (*p* < 0.05) rise in moisture content (MC) and a significant (*p* < 0.05) decline in protein, fat, and carbohydrate contents were noticed during both storage conditions. However, there was a non-significant increase (*p* > 0.05) in fiber and ash content. The respective MC of the GABA-rich beverage increased over 6 months from 84.78 to 86.21% and 84.78 to 86.88% when stored under refrigerated and ambient environments, respectively. However, the samples stored in refrigerated conditions showed less moisture absorption, which may be attributed to the difference in relative humidity between the two storage conditions. The protein content declined from 1.23 to 0.98% and 1.23 to 0.81% when stored under refrigerated and ambient environments, respectively. The protein content is thought to decrease with storage due to an increase in moisture and the Maillard reaction; however, more of an increase was noticed at ambient temperature. The respective fat content declined from 0.29 to 0.19% and 0.29 to 0.13% when stored under refrigerated and ambient environments. The slight variation might be due to the hydrolysis and oxidation of fat due to the increasing percentage of moisture content during storage. Similar results were demonstrated by Dhiman et al. [23] and Girija and Kamalasundari [24], and the authors attributed this variation to the moisture gained during storage. The increase in other nutrients (moisture, fiber, and ash contents) in the GABA-rich beverage resulted in the lowering of the carbohydrate content. Similarly, the carbohydrate content in a pomegranate- and vanilla-fortified synbiotic yogurt beverage was reduced from an initial value of 20% to 11.5% (*w*/*v*) after 9 weeks of storage [25].

### 3.6. Total and Reducing Sugars

The sugar concentration of the GABA-rich functional beverage declined with the storage period (Table 4 and Table 5). The total sugar concentration significantly (*p* < 0.05) declined from an initial value of 4.35 to 3.14%, and from 4.35 to 3.58%, for samples kept at ambient temperature and under refrigeration, respectively. Earlier studies also confirmed similar results. Coda et al. [26] produced a yogurt-like beverage using a composition of soy, cereals, and grape must and noticed that after 4 weeks of storage, the sugar amount had fallen by 90–92% of its initial value. Similarly, Yu et al. [27] recorded that after 10 days of storage at ambient temperature, the total sugar concentration of a probiotic Prunus mume puree was observed to reduce from an initial value of 0.1 to 0.066%. The reducing sugar concentration significantly (*p* < 0.05) increased from an initial value of 2.12 to 2.23%, and from 2.12 to 2.18%, for samples kept at ambient temperature and under refrigeration, respectively. A gradual rise in reducing sugars over the course of six months of storage may be the result of the conversion of polysaccharides to monosaccharides and the inversion of non-reducing sugars into reducing sugars. However, lesser variations were observed under refrigerated conditions due to the controlled environment. Similar reports were obtained by Dhiman et al. [23].

### 3.7. GABA and Gamma-Oryzanol

The GABA concentrations of the GABA-rich beverage samples stored under ambient temperature and refrigerated conditions are presented in Table 6 and Table 7. Initially, the samples had a GABA concentration of 42.12 mg/100 g. However, the GABA content in the beverage significantly decreased from 42.12 mg/100 g to 32.43 mg/100 g during storage at 25 °C. In contrast, when stored at 4 °C, the beverage showed a GABA content varying from 42.12 mg/100 g to 35.32 mg/100 g after 6 months. These results suggest that storing the beverage at low temperatures could prevent a reduction in GABA during storage. The degradation of GABA might occur at higher temperatures, leading to the production of gamma-butyrolactam due to the loss of water molecules, and this could be the possible mechanism behind its degradation, as reported previously by Contineanu et al. [28] and Khan et al. [29]. Since the storage temperature has an impact on the GABA concentration, the beverages should be kept at a low temperature to preserve this compound. Our results align with those of Zarei et al. [30] who reported that GABA production in a whey protein drink decreased during storage.

The concentrations of γ-oryzanol in the beverages stored under room-temperature and refrigerated conditions are presented in Table 6 and Table 7. Over the course of 6 months, there was a notable decrease in the γ-oryzanol levels across all samples. Initially, the beverage contained 52.73 mg/100 g of γ-oryzanol, which significantly decreased to 46.89 mg/100 g and 47.65 mg/100 g when stored under room-temperature and refrigerated conditions, respectively. This indicated that γ-oryzanol is stable under both ambient and refrigerated conditions. The stability of γ-oryzanol during storage could be attributed to its limited use in inhibiting oxidative deterioration [31].

### 3.8. Niacin Content

Table 6 and Table 7 illustrate the changes in niacin concentration in the GABA-rich beverage stored under room-temperature and refrigerated conditions. The findings revealed a decrease in niacin content in the GABA-rich beverage over an extended storage period. Initially, the beverage had a niacin content of 5.17 mg/100 g. Storage under both refrigerated and room-temperature conditions led to a respective reduction of approximately 24.95% and 39.65% in niacin content compared to their original levels. It was observed that storing the GABA-rich beverage under refrigerated conditions ensured safer storage. Additionally, niacin functions as an antioxidant to hinder lipid peroxidation, resulting in a gradual decrease in its concentration during storage [32]. Storing the beverage under refrigerated conditions could delay this loss in niacin concentration.

### 3.9. Total Phenolic Content

Table 6 and Table 7 present the impact of storage conditions (room temperature and refrigerated) on the total phenolic content (TPC) of the GABA-rich beverage over a 6-month storage period. Initially, the TPC of the samples was measured at 26.68 mg GAE/100 g. Generally, the TPC levels decreased after 6 months of storage, with the beverage stored at room temperature experiencing a 38.19% decrease in TPC during this period. Various factors influence the stability of phenolic compounds, including oxygen, temperature, pH, light, and duration of storage [33]. Piga et al. [34] suggested that elevated storage temperatures lead to the decomposition of phenolic components. Similarly, Pascual et al. [35] and Zhou et al. [36] noted the decomposition of phenolic compounds following storage at temperatures of 30 °C and 37 °C, respectively. However, by the end of the storage period, the beverage kept under refrigeration exhibited a TPC of 20.84 mg GAE/100 g, which was higher than that of the samples stored under room-temperature conditions (16.49 mg GAE/100 g). This suggests that lower temperatures can better preserve phenolic components during storage. These findings indicate that refrigerated conditions are more effective in maintaining maximum TPC in the stored GABA-rich beverage. Our results are in agreement with those of Shukla et al. [37], who reported that the TPC decreased in a sterilized mango-based dairy beverage during storage.

### 3.10. Antioxidant Capacities

The antioxidant potential could be due to several bioactive components, particularly phenolic acids, proanthocyanidins, anthocyanins, flavonoids, γ-oryzanol, tocotrienols, tocopherols, and phytic acid [33]. The antioxidant capacity of the GABA-rich beverage was evaluated using two methods: ferric-reducing antioxidant power and DPPH radical quenching capacity. Table 6 and Table 7 show a significant decrease in the antioxidant potential with the increase in storage time. The decrease in antioxidant capacity was more noticeable in beverage samples kept at ambient temperature compared to those stored under refrigeration. This difference was attributed to the increased activity of enzymes at higher temperatures, as stated in previous studies [38]. At the beginning of storage, the DPPH of the samples was found to be 74.23 μmol TE/100g, while the FRAP values were observed to be 13.05 μmol TE/100g (Table 6 and Table 7). The changes observed in the antioxidant levels during storage aligned with the fluctuations in the phenolic content. Besides the decomposition of phenolic compounds, other factors contributing to the notable decrease in antioxidant activity could include interactions between phenolic compounds and other constituents of the beverage during storage under both room-temperature and refrigerated conditions. Therefore, our findings show that storing the GABA-rich beverage in refrigerated conditions could effectively reduce the loss of antioxidants. The antioxidant capacity of the product could potentially help in preventing various health disorders, such as arthritis, cancer, diabetes, and atherosclerosis [39].

### 3.11. Total Plate Count

Initially, the total plate count in the GABA-rich beverage stored under both room-temperature and refrigerated conditions was undetectable (Table 6 and Table 7). However, over the storage period, the microbial population gradually increased. Notably, the storage environment and the type of packaging materials had a significant impact on microbial growth. Storage at room temperature led to higher microbial counts compared to refrigerated conditions, likely due to the elevated temperature. By the end of the storage period, the GABA-rich beverage stored under refrigerated conditions exhibited microbial counts of 0.42 CFU/mL, significantly lower than those stored at room temperature (1.49 CFU/mL). Nonetheless, throughout the 6-month storage period, the total plate count remained below 10^6^ CFU/mL, which is within the permissible limit according to FDA standards for beverages. Our findings are consistent with the results of a previous study [40].

### 3.12. Sensory Characteristics

**Figure 3:**
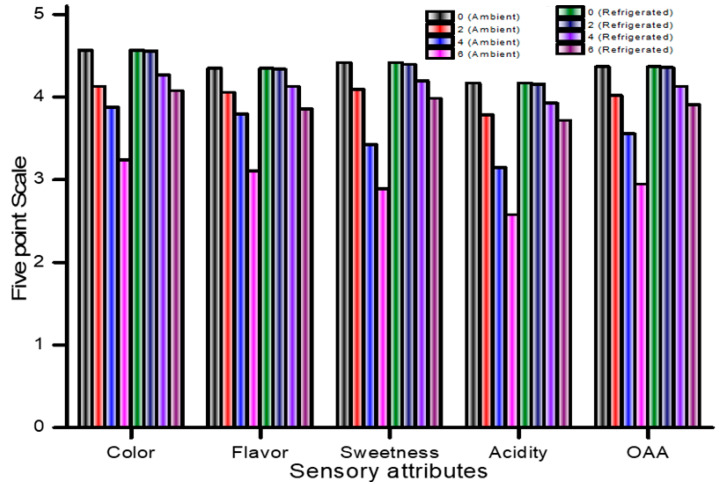
Sensory attributes of GABA-rich functional beverage during storage.

Differences in the sensory parameters of the GABA-rich beverage for both ambient and refrigerated storage conditions are presented in Figure 3. For the first two months of storage in a refrigerator, the beverage did not exhibit any sensory differences. However, after the second month, color and flavor differences were perceived. The color and flavor scores decreased with an increase in the storage period; however, a greater increase was detected when the samples were kept at room temperature, which indicated that a low storage temperature can retard the changes in flavor and color scores. Furthermore, the major characteristics of the product evaluated by the panelists during the sensory evaluation were sweetness and acidity, and the panelists detected changes in acidity during refrigerated storage. The study concluded that the beverage was suitable for consumption for up to 4 months at room temperature and 6 months when stored in the refrigerator, with slight acidification being detected by the sensory panels. These results were similar to those of Devi et al. [41] regarding whey-based fruit beverages stored under refrigeration.

## 4. Conclusions

Currently, there is no germinated rice-based beverage available on the market. Thus, this study aimed to optimize various ingredient levels to create an acceptable GABA-rich beverage. The total GABA content in the developed beverage was measured at 42.12 mg/100 g, which corresponds to 14% of the recommended daily intake of GABA (300 mg/day) according to FAO/WHO guidelines. The consumption of 250 mL of this beverage would provide approximately 105.3 mg of GABA, fulfilling around 35% of the recommended daily allowance. Consequently, the findings of this study present a new opportunity for food industries to utilize germinated brown rice for producing functional beverages. Conclusively, development of a GABA-rich beverage using germinated brown rice opens up a new avenue for food industries, providing a product with significant potential to contribute to daily GABA intake.

## Figures and Tables

**Table 1:** Central composite rotatable design for the independent variables ^1^.

Independent Variables	Code	Levels in Coded Form				
		−2	−1	0	+1	+2
Powdered sugar (g)	X_1_	10	20	30	40	50
Chocolate powder (g)	X_2_	15	20	25	30	35
Sodium carboxymethyl cellulose (g)	X_3_	0.2	0.3	0.4	0.5	0.6
GBR (g)/Water (mL)	X_4_	150:350	190:380	205:410	220:440	235:470

^1^ GBR, germinated brown rice.

**Table 2:** Analysis of variance for the fitted models.

	Source	Coefficient	Sum of Square	Degree of Freedom	Mean Square	F Value	*p*-Value
Overall acceptability	Residual		1.66	15	0.1109		
	Lack of fit		1.49	10	0.1489	4.25	<0.0001
	Pure error		0.1750	5	0.0350		
	Total		25.31	29			
	R^2^	0.9342					
	Adj-R^2^	0.8729					
	CV	4.76					
	Adequate precision	16.0634					
Total soluble solids (°Brix)	Residual		1.88	15	0.1254		
	Lack of fit		1.45	10	0.1452	1.70	<0.0001
	Pure error		0.4283	5	0.0857		
	Total		88.87	29			
	R^2^	0.9788					
	Adj-R^2^	0.9591					
	CV	3.59					
	Adequate precision	29.2878					

**Table 3:** Effect of independent variables on dependent variables of GABA-rich beverage derived from germinated brown rice ^1^.

S. No.	Sugar (g)	Chocolate (g)	SCMC (g)	GBR (g)/Water (mL)	OAA	TSSs (°Brix)
1	10	25	0.4	205:410	1.5	6.8
2	30	35	0.4	205:410	2.6	10.7
3	30	25	0.4	205:410	2.9	9.3
4	20	30	0.5	190:380	1.8	8.6
5	20	30	0.5	220:440	1.9	8.5
6	40	30	0.3	190:380	4	12.3
7	20	20	0.3	220:440	2.4	7.2
8	30	25	0.6	205:410	2.7	10.5
9	40	30	0.3	220:440	4.2	12.1
10	30	25	0.4	205:410	3	9.4
11	20	20	0.5	220:440	2.2	7.8
12	30	25	0.4	205:410	3.1	9.5
13	40	20	0.3	190:380	4.3	11.5
14	50	25	0.4	205:410	4.1	12.9
15	20	30	0.3	220:440	1.7	8.2
16	40	20	0.5	220:440	4.6	11.7
17	20	20	0.3	190:380	2.3	7.5
18	30	25	0.4	205:410	3.2	9.6
19	30	25	0.2	205:410	3.6	9.1
20	30	25	0.4	235:470	3.7	9.7
21	40	30	0.5	190:380	3.8	12.6
22	30	25	0.4	205:410	3.3	9.8
23	30	15	0.4	205:410	3.5	8.9
24	40	20	0.3	220:440	4.5	11.2
25	30	25	0.4	175:350	2.8	9.9
26	40	20	0.5	190:380	4.2	11.9
27	40	30	0.5	220:440	3.9	12.5
28	30	25	0.4	205:410	3.4	10.1
29	20	30	0.3	190:380	1.6	8.3
30	20	20	0.5	190:380	2.1	7.9

^1^ SCMC, sodium carboxymethyl cellulose; GABA, γ-aminobutyric acid; GBR, germinated brown rice; OAA, overall acceptability; TSSs, total soluble solids. Sugar and chocolate were added in powder form.

**Table 4:** Changes in the physicochemical composition of GABA-rich functional beverage during ambient storage ^1^.

Parameters	Ambient			
	0	2	4	6
Moisture (%)	84.78 ± 1.21 ^b^	84.95 ± 1.27 ^b^	85.72 ± 1.32 ^ab^	86.88 ± 1.44 ^a^
Protein (%)	1.23 ± 0.18 ^b^	1.16 ± 0.16 ^b^	1.08 ± 0.13 ^b^	0.81 ± 0.09 ^a^
Fat (%)	0.29 ± 0.09 ^b^	0.25 ± 0.07 ^b^	0.19 ± 0.06 ^ab^	0.13 ± 0.04 ^a^
Fiber (%)	0.37 ± 0.11 ^a^	0.39 ± 0.12 ^a^	0.42 ± 0.14 ^a^	0.47 ± 0.16 ^a^
Ash (%)	0.12 ± 0.05 ^a^	0.14 ± 0.06 ^a^	0.16 ± 0.07 ^a^	0.20 ± 0.08 ^a^
Carbohydrates (%)	13.21 ± 0.27 ^c^	13.11 ± 0.25 ^c^	12.43 ± 0.24 ^b^	11.51 ± 0.22 ^a^
Total sugars (%)	4.35 ± 0.41 ^c^	4.17 ± 0.37 ^bc^	3.66 ± 0.34 ^ab^	3.14 ± 0.29 ^a^
Reducing sugars (%)	2.12 ± 0.09 ^a^	2.15 ± 0.11 ^a^	2.19 ± 0.13 ^a^	2.23 ± 0.14 ^b^

^1^ Values are presented as mean ± standard deviation. Mean values with different superscripts within rows differ significantly (*p* < 0.05). GABA, γ-aminobutyric acid.

**Table 5:** Changes in physicochemical composition of GABA-rich functional beverage during refrigerated storage ^1^.

Parameters	Refrigerated			
	0	2	4	6
Moisture (%)	84.78 ± 1.21 ^b^	84.84 ± 1.25 ^b^	85.36 ± 1.29 ^ab^	86.21 ± 1.37 ^a^
Protein (%)	1.23 ± 0.18 ^b^	1.19 ± 0.15 ^b^	1.14 ± 0.11 ^ab^	0.98 ± 0.07 ^a^
Fat (%)	0.29 ± 0.09 ^b^	0.27 ± 0.08 ^b^	0.23 ± 0.06 ^b^	0.19 ± 0.05 ^a^
Fiber (%)	0.37 ± 0.11 ^a^	0.38 ± 0.13 ^a^	0.41 ± 0.15 ^a^	0.45 ± 0.18 ^a^
Ash (%)	0.12 ± 0.05 ^a^	0.13 ± 0.05 ^a^	0.15 ± 0.06 ^a^	0.18 ± 0.07 ^a^
Carbohydrates (%)	13.21 ± 0.27 ^c^	13.19 ± 0.26 ^c^	12.71 ± 0.24 ^b^	11.99 ± 0.21 ^a^
Total sugars (%)	4.35 ± 0.41 ^b^	4.24 ± 0.39 ^b^	3.89 ± 0.34 ^ab^	3.58 ± 0.31 ^a^
Reducing sugars (%)	2.12 ± 0.09 ^a^	2.13 ± 0.11 ^a^	2.16 ± 0.12 ^a^	2.18 ± 0.14 ^b^

^1^ Values are presented as mean ± standard deviation. Mean values with different superscripts within rows differ significantly (*p* < 0.05). GABA, γ-aminobutyric acid.

**Table 6:** Changes in bioactive compounds, antioxidant properties, and total plate count of GABA-rich functional beverage during ambient storage ^1^.

Parameters	Ambient			
	0	2	4	6
GABA (mg/100 g)	42.12 ± 0.63 ^d^	39.54 ± 0.57 ^c^	36.67 ± 0.53 ^b^	32.43 ± 0.48 ^a^
γ-oryzanol (%)	52.73 ± 1.56 ^c^	51.17 ± 1.48 ^bc^	49.21 ± 1.39 ^ab^	46.89 ± 1.24 ^a^
Niacin (%)	5.17 ± 0.14 ^d^	4.41 ± 0.13 ^c^	4.03 ± 0.11 ^b^	3.12 ± 0.08 ^a^
TPC (mg GAE/100 g)	26.68 ± 1.56 ^c^	24.53 ± 1.48 ^c^	21.37 ± 1.41 ^b^	16.49 ± 1.32 ^a^
DPPH (µmol TE/100 g)	74.23 ± 2.37 ^c^	72.03 ± 2.09 ^bc^	70.52 ± 1.93 ^b^	66.34 ± 1.62 ^a^
FRAP (µmol TE/100 g)	13.05 ± 0.38 ^d^	12.13 ± 0.34 ^c^	10.41 ± 0.29 ^b^	8.24 ± 0.24 ^a^
Total plate count (CFU/mL)	ND	0.58 ± 0.06 ^a^	0.87 ± 0.07 ^b^	1.49 ± 0.09 ^c^

^1^ Values are presented as mean ± standard deviation. Mean values with different superscripts within rows differ significantly (*p* < 0.05). GABA, γ-aminobutyric acid; TPC, total phenolic content; GAE, gallic acid equivalent, DPPH, 2,2-diphenyl-1-picrylhydrazyl; FRAP, ferric-reducing antioxidant power; TE, Trolox equivalent; CFU, colony-forming unit; ND: not detected.

**Table 7:** Changes in bioactive compounds, antioxidant properties, and total plate count of GABA-rich functional beverage during refrigerated storage ^1^.

Parameters	Refrigerated			
	0	2	4	6
GABA (mg/100 g)	42.12 ± 0.63 ^d^	40.65 ± 0.58 ^c^	38.75 ± 0.54 ^b^	35.32 ± 0.51 ^a^
γ-oryzanol (%)	52.73 ± 1.56 ^c^	51.86 ± 1.49 ^bc^	49.71 ± 1.41 ^ab^	47.65 ± 1.27 ^a^
Niacin (%)	5.17 ± 0.14 ^d^	4.74 ± 0.13 ^c^	4.32 ± 0.09 ^b^	3.88 ± 0.07 ^a^
TPC (mg GAE/100 g)	26.68 ± 1.56 ^c^	25.42 ± 1.49 ^bc^	23.16 ± 1.43 ^ab^	20.84 ± 1.35 ^a^
DPPH (µmol TE/100 g)	74.23 ± 2.37 ^b^	73.44 ± 2.15 ^b^	72.61 ± 1.96 ^b^	71.56 ± 1.74 ^a^
FRAP (µmol TE/100 g)	13.05 ± 0.38 ^c^	12.72 ± 0.36 ^b^	11.57 ± 0.30 ^ab^	10.11 ± 0.27 ^a^
Total plate count (CFU/mL)	ND	ND	0.19 ± 0.03^a^	0.42 ± 0.06^b^

^1^ Values are presented as mean ± standard deviation. Mean values with different superscripts within rows differ significantly (*p* < 0.05). GABA, γ-aminobutyric acid; TPC, total phenolic content; GAE, gallic acid equivalent, DPPH, 2,2-diphenyl-1-picrylhydrazyl; FRAP, ferric-reducing antioxidant power; TE, Trolox equivalent; CFU, colony-forming unit; ND: not detected.

## Data Availability

The original contributions presented in the study are included in the article, further inquiries can be directed to the corresponding authors.
